# Intraspecific size variation in planktonic foraminifera cannot be consistently predicted by the environment

**DOI:** 10.1002/ece3.6792

**Published:** 2020-09-28

**Authors:** Marina C. Rillo, C. Giles Miller, Michal Kucera, Thomas H. G. Ezard

**Affiliations:** ^1^ Ocean and Earth Science National Oceanography Centre Southampton University of Southampton Waterfront Campus Southampton UK; ^2^ Department of Earth Sciences Natural History Museum London UK; ^3^ Center for Marine Environmental Sciences University of Bremen Bremen Germany

**Keywords:** biogeography of traits, body size, ecological optima, macroecology, morphometrics, natural history collections, zooplankton

## Abstract

The size structure of plankton communities is an important determinant of their functions in marine ecosystems. However, few studies have quantified how organism size varies within species across biogeographical scales. Here, we investigate how planktonic foraminifera, a ubiquitous zooplankton group, vary in size across the tropical and subtropical oceans of the world. Using a recently digitized museum collection, we measured shell area of 3,799 individuals of nine extant species in 53 seafloor sediments. We first analyzed potential size biases in the collection. Then, for each site, we obtained corresponding local values of mean annual sea‐surface temperature (SST), net primary productivity (NPP), and relative abundance of each species. Given former studies, we expected species to reach largest shell sizes under optimal environmental conditions. In contrast, we observe that species differ in how much their size variation is explained by SST, NPP, and/or relative abundance. While some species have predictable size variation given these variables (*Trilobatus sacculifer, Globigerinoides conglobatus, Globigerinella siphonifera, Pulleniatina obliquiloculata, Globorotalia truncatulinoides*), other species show no relationships between size and the studied covariates (*Globigerinoides ruber*, *Neogloboquadrina dutertrei*, *Globorotalia menardii, Globoconella inflata*). By incorporating intraspecific variation and sampling broader geographical ranges compared to previous studies, we conclude that shell size variation in planktonic foraminifera species cannot be consistently predicted by the environment. Our results caution against the general use of size as a proxy for planktonic foraminifera environmental optima. More generally, our work highlights the utility of natural history collections and the importance of studying intraspecific variation when interpreting macroecological patterns.

## Graphical abstract

Using a recently digitized museum collection, we investigate how planktonic foraminifera species vary in size across biogeographical scales. We found that the relationships between shell size and the explanatory variables (temperature, net primary productivity, and local relative abundance) differ among the nine studied species, contrary to the idea that species are largest at their environmental optima.

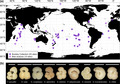

## 1 INTRODUCTION

The size structure of plankton communities is an important determinant of the functions they realize in marine ecosystems, such as the energy transfer along the marine food web and the exchange of carbon between the atmosphere and the deep ocean (Barton et al., 2013; Litchman, Ohman, & Kiørboe, 2013). So far, most studies have focused on size distributions of assemblages (irrespective of species) or interspecific (among‐species) variation instead of intraspecific (within‐species) variation (Sommer, Peter, Genitsaris, & Moustaka‐Gouni, 2017). Intraspecific variation can affect trophic interactions (Des Roches et al., 2018) and influence species' responses to environmental change (Mousing et al., 2017). Thus, by ignoring intraspecific variation, we have an incomplete understanding of the functions different plankton species perform in the ecosystem.

Planktonic foraminifera are an interesting group for studying intraspecific size variation. They are unicellular zooplankton that occur across the world's oceans at low diversities (48 currently recognized species; Siccha & Kucera, 2017) and produce calcium carbonate tests (hereafter “shells”, see Kucera, 2007). Upon death, their shells sink and accumulate on the ocean floor, playing a key role in the ocean carbon cycle (Schiebel, 2002) and, under favorable sedimentary conditions, yielding a remarkably complete fossil record (Kucera, 2007). The abundance of their shells preserved in marine sediments allows estimates of size variation on a global scale (Schmidt, Renaud, Bollmann, Schiebel, & Thierstein, 2004) and over geological time scales including past environmental changes (Schmidt, Thierstein, Bollmann, & Schiebel, 2004). Thus, planktonic foraminifera can help elucidate the functional role of intraspecific size variation in marine ecosystems across space and in time. However, a quantification of individual size variation across large biogeographical ranges is missing, limiting our understanding of what controls their within‐species size variation.

Planktonic foraminifera grow by sequential addition of chambers until reproduction, when the cell dies (semelparity; Hemleben, Spindler, & Anderson, 1989). Seawater temperature affects their growth directly through biochemical reaction rates or indirectly by influencing oxygen availability and the abundance of prey and symbionts (Caron, Faber, & Bé, 1987; Bijma, Faber, & Hemleben, 1990; Bijma, Hemleben, Oberhaensli, & Spindler, 1992; Burke et al. 2018; Lombard, Erez, Michel, & Labeyrie, 2009; Takagi et al., 2019). On a global scale, planktonic foraminifera assemblages increase in size with increasing sea‐surface temperature, and the largest species occur in the tropics (Schmidt, Renaud, et al., 2004). This pattern is opposite to the negative relationship between size and temperature observed in other protist and plankton groups (the “temperature–size rule,” Atkinson, Ciotti, & Montagnes, 2003; Barton et al., 2013; Sommer et al., 2017). Planktonic foraminifera are difficult to culture, so many aspects of their life history are still unknown, including the processes underlying their morphological variation (Davis et al., 2020). Their generation times seem to be constrained by a synchronous sexual reproduction (Bijma, Erez, & Hemleben, 1990; Jonkers, Reynolds, Richey, & Hall, 2015; Venancio et al., 2016), and the final shell size possibly determines the number of gametes released during gametogenesis (Hemleben et al., 1989). Consequently, larger sizes have been often associated with higher reproductive success (e.g., Grigoratou et al., 2019), although a quantification of this relationship is absent and the recent observation of asexual reproduction in the group (Davis et al., 2020; Takagi, Kurasawa, & Kimoto, 2020) might change this interpretation. In the fossil record, there is evidence that species decrease in average size before going extinct (Brombacher, Wilson, Bailey, & Ezard, 2017; Wade & Olsson, 2009), which suggests that smaller sizes are related to suboptimal and stressful conditions.

Previous studies have looked at size variation within modern species of planktonic foraminifera and found that maximum shell size often coincides with maximum relative abundance, and occurs at specific optimum temperatures (“optimum‐size hypothesis”; Hecht, 1976; Kahn, 1981; Kennett, 1976; Malmgren & Kennett, 1976, 1977; Moller, Schulz, & Kucera, 2013; Schmidt, Renaud, et al., 2004; see Be, Harrison, & Lott, 1973 for an exception). The local abundance of planktonic foraminifera species is usually estimated by counting dead assemblages from ocean floor sediments (Siccha & Kucera, 2017). This methodology yields species' relative abundances (relative to the co‐occurring species in the sample) instead of absolute abundances, and has the advantage of averaging out short‐term fluctuations that might blur biogeographical patterns (Kidwell & Tomasovych, 2013). Most of the studies supporting the optimum‐size hypothesis focused on sediment samples collected within a single oceanic basin, and thus a limited part of each species' biogeographical range. The exception is the global study of Schmidt, Renaud, et al. (2004), who analyzed 69 Holocene samples worldwide. Schmidt, Renaud, et al. (2004) were concerned with size variation of assemblages and only taxonomically identified a small fraction of the measured individuals. Nevertheless, they showed a tight 1:1 relationship between the temperatures at which a species reaches its largest size and its highest relative abundance (Schmidt, Renaud, et al., 2004), often cited as support for the optimum‐size hypothesis. However, such a 1:1 species‐level relationship can also emerge if the analyzed species have different thermal niches regardless of the direct relationship between size and abundance within species. Because of the different thermal niches, the species‐level relationship between the temperatures of largest sizes and highest relative abundances will be always scattered around the 1:1 relationship, with tighter relationships emerging when the thermal niches are narrower. Thus, the optimum‐size hypothesis requires validation by new, population‐level observations. If species reach largest shell sizes at optimum temperatures, this pattern should be evident among their populations.

Another environmental factor that may influence planktonic foraminifera size distributions is food availability. Higher feeding frequency has been shown experimentally to result in faster cell growth and larger final shell size (Be, Caron, & Anderson, 1981; Bijma et al., 1992; Takagi, Kimoto, Fujiki, & Moriya, 2018). Planktonic foraminifera have diverse trophic strategies. Species can be heterotrophic or show different levels of photosymbiosis (Hemleben et al., 1989; Takagi et al., 2019), but even persistent (obligatory) photosymbiosis cannot be the only form of daily nutrition (Takagi et al., 2018). Planktonic foraminifera are omnivorous, preying on other plankton including diatoms, dinoflagellates, ciliates, and copepods (Hemleben et al., 1989). Thus, phytoplankton are part of their diet and also attract other zooplankton predated by them. If net primary productivity can be used as a proxy for planktonic foraminifera food availability, we expect intraspecific size variation to correlate positively with primary productivity. However, this relationship likely depends on the species' trophic strategy (Lombard, Erez, et al., 2009) and has never been quantified on a biogeographical scale.

Here, we built a new morphometric dataset of species‐resolved planktonic foraminifera in the tropical and subtropical world oceans. We quantify the relationship between within‐species size variation and mean annual sea‐surface temperature (SST), mean annual net primary productivity (NPP), and relative abundance, plus the interaction between SST and NPP. Organism size was assessed as the cross‐sectional area of the shell. We expect size to (a) increase with increasing SST for tropical species and (b) reach largest values at intermediate SST for transitional species (Schmidt, Lazarus, Young, & Kucera, 2006). We also expect (c) species to reach larger sizes where there are more resources available (i.e., higher NPP). As SST and NPP correlate in the open ocean (Schmidt et al., 2006), these two variables might interact and jointly predict more of the observed size variation than when tested alone. Lastly, the optimum‐size hypothesis predicts (d) a positive relationship between size and local relative abundance (Hecht, 1976).

## 2 MATERIAL AND METHODS

**FIGURE 1:**
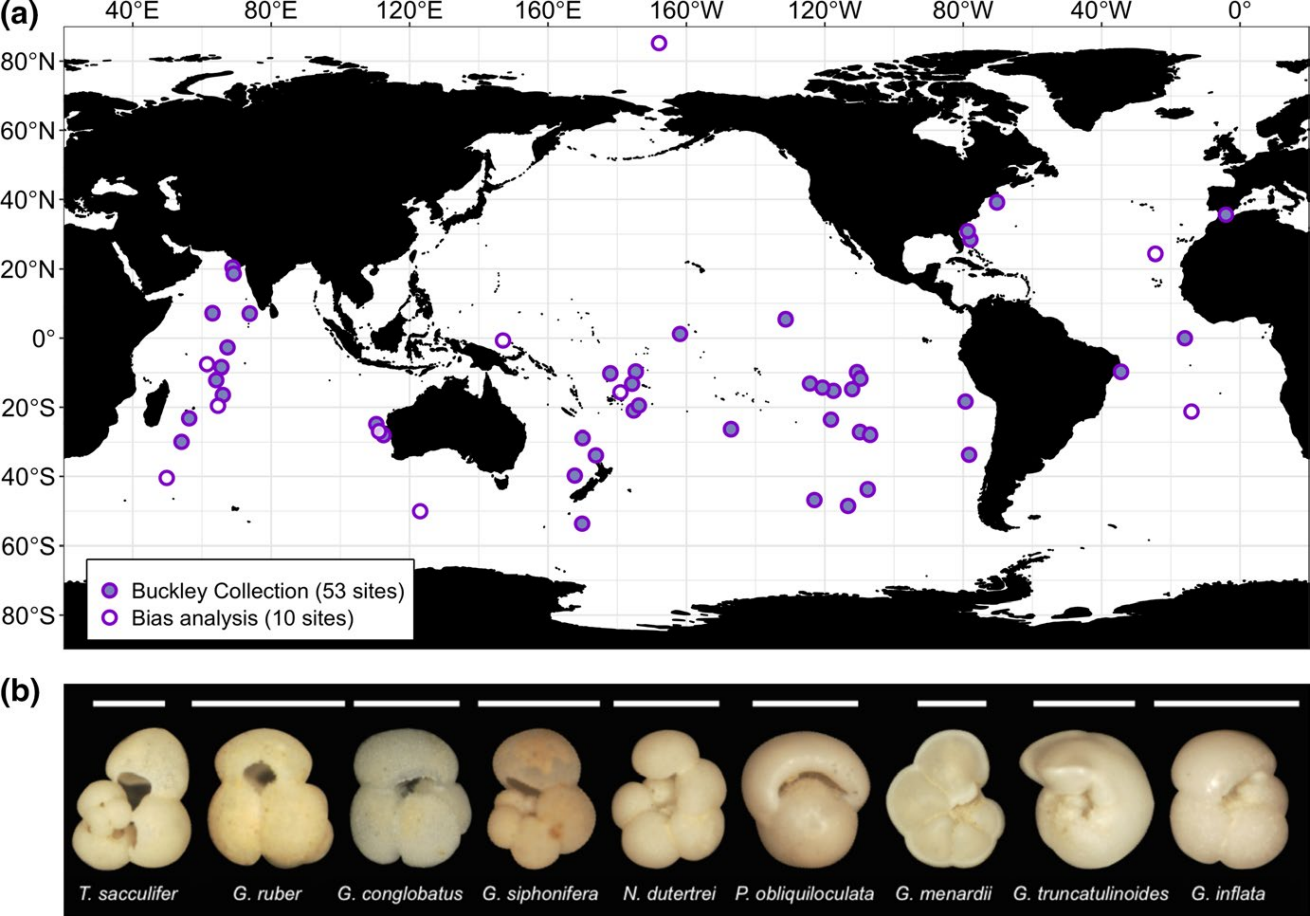
(a) The geographic distribution of the samples in the morphometric dataset. Each dot on the map includes data on planktonic foraminifera shell sizes, species' relative abundances, and mean annual values of sea‐surface temperature and net primary productivity. The nonsolid dots represent the ten samples that were used in the bias analysis of the Buckley Collection; the sample above 80°N was used only in the bias analysis. (b) A representative specimen from the Buckley Collection for each species analyzed. White bars represent 500 μm. From left to right: *Trilobatus sacculifer* (NHMUK museum number: ZF6250 (30)), *Globigerinoides ruber* (ZF6269 (1)), *Globigerinoides conglobatus* (ZF6318 (9)), *Globigerinella siphonifera* (ZF6667 (2)), *Neogloboquadrina dutertrei* (ZF6404 (7)), *Pulleniatina obliquiloculata* (ZF6631 (14)), *Globorotalia menardii* (ZF5778 (4)), *Globorotalia truncatulinoides* (ZF5839 (14)), and *Globoconella inflata* (ZF5969 (32))

Our morphometric dataset was extracted from the Henry Buckley Collection of Planktonic Foraminifera (Rillo et al., 2016), held at the Natural History Museum in London, UK (NHMUK). We measured the shell area of 3,799 individuals from nine species across 53 sites worldwide (Figure 1). For each sampled site, we obtained corresponding data on the relative abundance of each species and the mean annual values of SST and NPP. All data visualization and analyses were performed in R (version 3.3.3, R Core Team, 2017).

### 2.1 Study sites and samples

To amass the Henry Buckley Collection of Planktonic Foraminifera, Henry Buckley sampled 122 marine sediments from the NHMUK Ocean‐Bottom Deposits Collection (OBD, Miller, 2018), which were collected by historical marine expeditions between 1873 and 1965 (Table S1) (Rillo et al., 2016). Sample processing usually consists of washing the sediment and dry sieving it over a 150 μm sieve, and then sampling the coarser fraction for planktonic foraminifera (Al‐Sabouni, Kucera, & Schmidt, 2007). From the 122 samples processed by Buckley, we selected those that contained only extant species within the upper 15 cm of sediment and included at least one of the nine studied species (Table 1). This resulted in 53 study sites predominantly in the tropical and subtropical regions of the Pacific, Indian, and Atlantic oceans (Figure 1a).

**TABLE 1:** Overview of the morphometric data extracted from the Buckley Collection

Species	*N* (ind)	*N* (site)	Avg. *N* (ind/site)	Mounting position
*Trilobatus sacculifer*	674	38	15	Umbilical or spiral
*Globigerinoides ruber*	481	39	10	Umbilical or spiral
*Globigerinoides conglobatus*	345	38	8	Umbilical
*Globigerinella siphonifera*	244	37	5	Umbilical or spiral
*Neogloboquadrina dutertrei*	321	30	9	Umbilical
*Pulleniatina obliquiloculata*	295	32	8.5	Edge
*Globorotalia menardii*	665	29	16	Umbilical or spiral
*Globorotalia truncatulinoides*	311	30	8.5	Umbilical
*Globoconella inflata*	463	19	17	Umbilical

Note

3,799 specimens were measured in total. Columns: species names; number of specimens (individuals) measured; number of sites per species (populations); median number of individuals per site; mounting position is the orientation in which the species was measured.

We determined the water depth for each site by matching the collection's reported latitudes and longitudes to the ETOPO1 database hosted at the National Oceanic and Atmospheric Administration website (Amante & Eakins, 2009) using a 2 arc‐minute grid resolution (R package *marmap* version 0.9.5; Pante & Simon‐Bouhet, 2013). Water depth ranged from 746 to 5,153 meters below sea level (median 3,296 m). Ten of the 53 samples in our dataset come from sediments prone to dissolution (i.e., waters deeper than 4,000 m for newly sedimented shells; Berger & Piper, 1972). Although dissolution may affect species size distributions (as smaller individuals are more prone to dissolution; Kennett, 1976), we found no evidence that water depth is related to the size variation observed in our data (Table S2).

### 2.2 Shell‐size data

We measured the shell area of the planktonic foraminifera species most commonly represented in the Buckley Collection (Rillo et al., 2016). *Neogloboquadrina pachyderma* was not included because this species was mounted on the slides together with *N. incompta*. Specimens were imaged using a Zeiss Axio Zoom V16 microscope and ZEN software at a resolution of 2.58 μm × 2.58 μm per pixel. Individual size was estimated based on the cross‐sectional area of the two‐dimensional image of the specimen using a bespoke macro in Image‐Pro Premier (version 9.1) that automatically recognizes each specimen and measures its area. Brombacher, Elder, Hull, Wilson, and Ezard (2018) showed that the cross‐sectional area of planktonic foraminifera shells can provide a consistent proxy for shell volume. Henry Buckley mounted most specimens on the slides in a standard, taxonomically relevant orientation (Figure 1b and Table 1). Brombacher, Wilson, and Ezard (2017) quantified the reproducibility of shell area measurements and concluded that this two‐dimensional metric is highly consistent across slight deviations in mounting orientation. We avoided remounting the slides to preserve the Buckley Collection, but individuals with a different orientation or dubious taxonomic identification were excluded from the analysis. In this way, each species had the same orientation in all the measurements, allowing us to assess size variation consistently within species.

**FIGURE 2:**
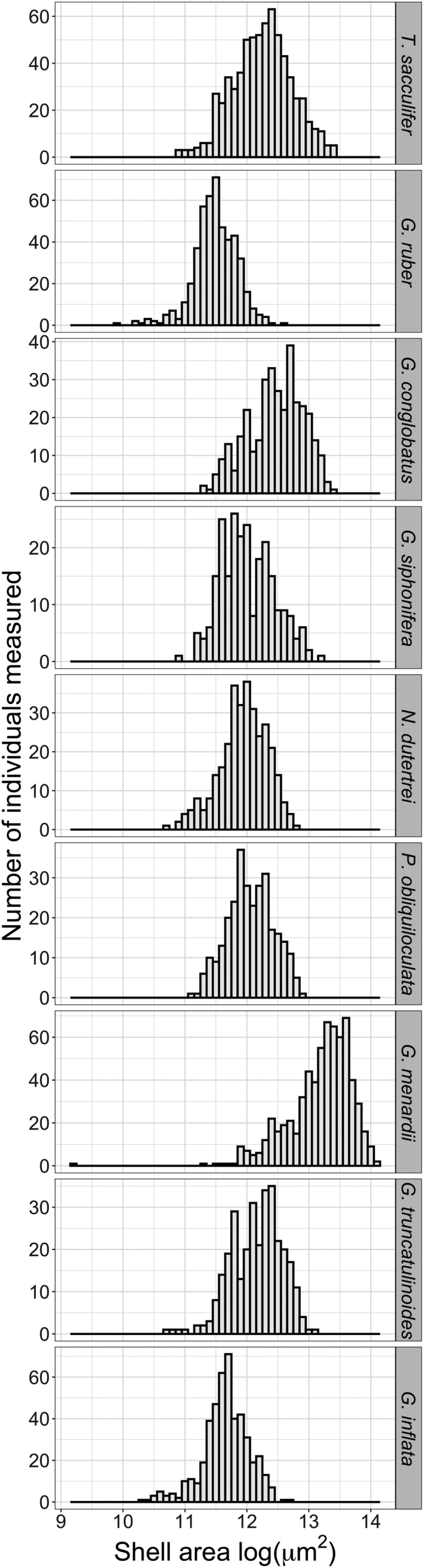
Size histograms for each species of planktonic foraminifera present in the morphometric dataset. Size was assessed as the cross‐sectional area of the shell and log‐transformed (natural logarithm). From top to bottom: *T. sacculifer*, *G. ruber*, *G. conglobatus*, *G. siphonifera, N. dutertrei*, *P. obliquiloculata*, *G. menardii, G. truncatulinoides,* and *G. inflata*. See also Table 1

In total, we measured 3,799 specimens from nine species (Figure 2). Each species is represented by at least 244 specimens in the morphometric dataset (Table 1). Ideally, more individuals would have been measured; however, the taxonomic identification, imaging, and measurement of specimens (besides sample processing) are time‐consuming steps to build a global, species‐resolved morphometric dataset. In the future, automated species identification methods (e.g. Hsiang et al., 2019) will greatly facilitate the compilation of larger datasets.

### 2.3 Bias analysis

**FIGURE 3:**
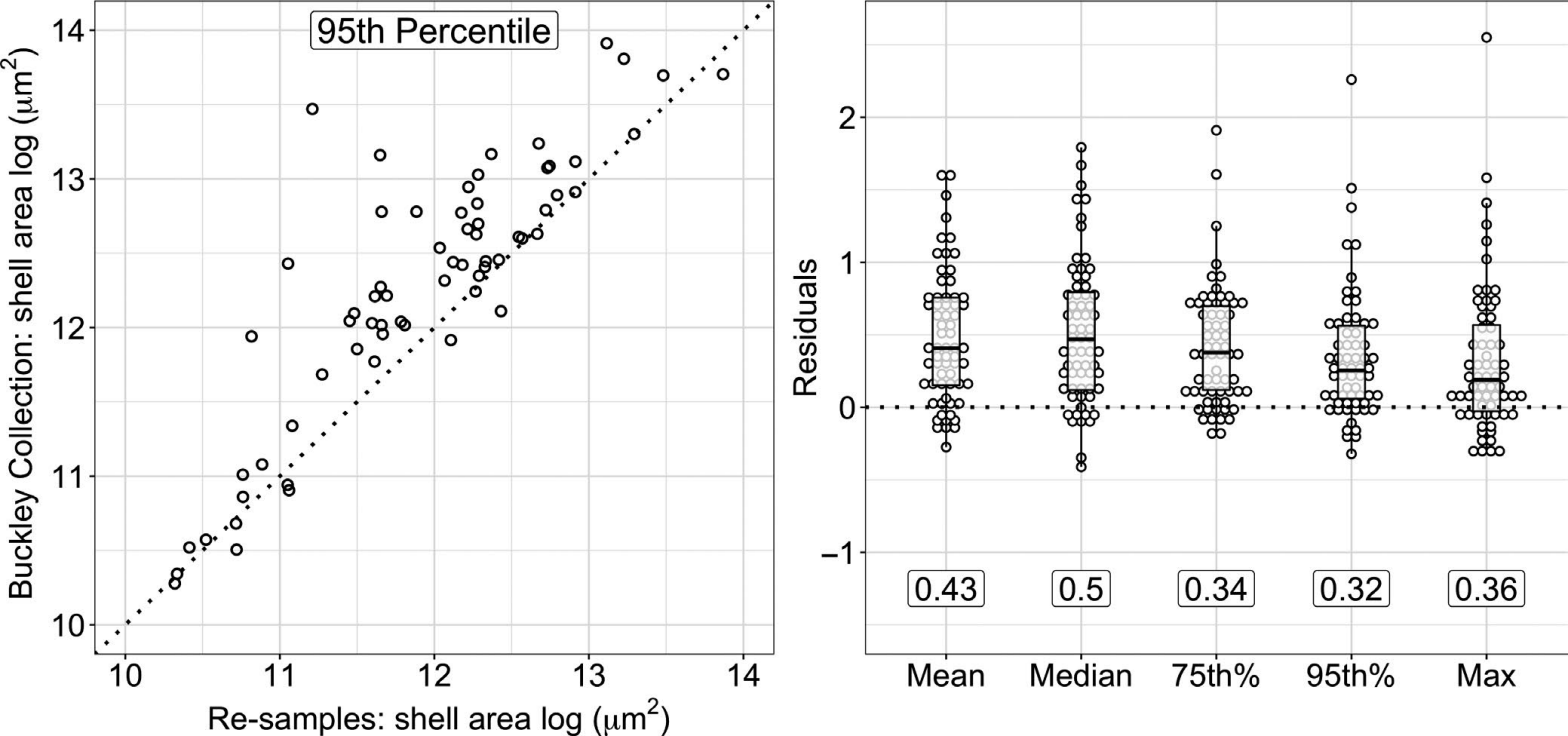
Bias analysis of planktonic foraminifera size distributions from the Buckley Collection. Size was assessed as the cross‐sectional area of the shell and log‐transformed (natural logarithm). (a) Size distributions from the Buckley Collection against our resampled samples. Each dot represents the 95th percentile of the population size distribution. Dotted line represents the identity function (1:1 relationship). (b) Residuals of the 1:1 relationship for five distribution metrics: mean, median, 75th percentile, 95th percentile, and maximum value. Numbers indicate the mean squared error (*MSE*) of each metric. The 95th percentile has the lowest *MSE* of the five metrics. See also Appendix S1

The Buckley Collection could exhibit a collector effort bias, typically believed to occur toward larger specimens. To assess this potential bias, we resampled ten original bulk sediments from the OBD Collection (nonsolid dots in Figure 1a, Table S3) that Buckley had used to amass his collection. We processed these ten samples (see Appendix S1) and mounted species‐specific slides to extract shell‐size data in the same way as for the original Buckley Collection (described above). We then compared the shell‐size distributions between the Buckley Collection samples and our resampled samples. This comparison included 2,873 individuals (1,049 from the Buckley Collection and 1,824 from the samples picked by us), across 65 populations from 20 species collected from the 10 sites. We log‐transformed the shell area data and calculated the mean, median, 75th percentile, 95th percentile, and maximum value of each population shell‐size distribution. We then regressed each of these five metrics of the Buckley Collection against the metrics of our resampled data (e.g., Figure 3a) and calculated the residuals assuming 1:1 correspondence. The residuals of the regressions are predominantly positive (Figure 3b), indicating that the Buckley Collection has a consistent collector bias toward large specimens. As Henry Buckley personally carried out all the sample processing, isolation of foraminiferal specimens and their identification, the collector biases in his collection are likely to be systematic for within‐species comparisons.

The mean squared error is lowest for the 95th percentile (Figure 3b), meaning that this metric is the most representative population‐level metric of the Buckley Collection. The robustness of the 95th percentile of size distributions has also been documented by Schmidt, Renaud, et al. (2004); it is less sensitive to single outliers than the maximum value, and to representative sampling at the lower end of the size distribution than the mean and median values. Accordingly, we used the 95th percentiles of the population shell‐size distributions as the dependent variable to investigate the covariates of planktonic foraminifera intraspecific size variation.

### 2.4 Sea‐surface temperature data

We compiled mean annual values of SST from the World Ocean Atlas 2013 (WOA13, 0 m depth, Locarnini et al., 2013) for each morphometric sample by matching its unique latitude and longitude coordinates to the nearest WOA13 1^◦^ grid point (approximately 111 km at the equator). The distances between the datasets were calculated using the World Geodetic System of 1984 (WGS 84) and R package *geosphere* (version 1.5‐7; Hijmans, 2015). We used SST data from the earliest decade available in the WOA13 database, resulting in SST data averaged for the years between 1955 and 1964. We chose this time period because the latest historical expedition associated with our morphometric dataset sailed in 1965 (Table S1).

### 2.5 Net primary productivity data

We compiled mean annual values of NPP from the Ocean Productivity website (http://www.science.oregonstate.edu/ocean.productivity/). We selected the SeaWiFS estimates (based on the Eppley variation of the VGPM algorithm; Behrenfeld & Falkowski, 1997) because they provide the earliest NPP data (starting in late 1997). We matched each morphometric sample coordinate to its nearest NPP sample as described for SST. The median distance between the datasets was 15 km. We considered only full years of NPP data collection, from January 1998 until December 2007.

### 2.6 Relative abundance data

To test for the relationship between population shell size and abundance (Hecht, 1976; Schmidt, Renaud, et al., 2004), we extracted assemblage composition data from the ForCenS database (Siccha & Kucera, 2017). Henry Buckley did not identify all specimens in each sample, preventing the assessment of species abundances from his collection. The ForCenS database is a synthesis of planktonic foraminifera assemblage counts from surface sediment samples with 4,205 records from unique sites worldwide, each with corresponding information on species' relative abundance. We retrieved species relative abundance data for each morphometric sample by matching its coordinates to its nearest neighbor in the ForCenS database as described for SST. The median distance between the datasets was 106 km.

The spatial arrangement of shells on the seafloor is affected during settling by subsurface currents (Berger & Piper, 1972). Recent models estimate that settling foraminiferal shells can travel a maximum distance of 300 km in regions with large horizontal velocities (e.g., along the equator, in the western boundary currents, and in the Southern Ocean; Van Sebille et al., 2015). To account for this postmortem spatial variation of foraminiferal abundance on the seafloor, we retrieved ForCenS abundance data within a 300 km radius distance of each morphometric sample coordinate, which would be the maximum error according to Van Sebille et al. (2015). We then calculated the median relative abundance of each species based on all ForCenS samples that fell within 300 km of the morphometric sample. The analysis considering all ForCenS samples within 300 km distance produced consistent results compared to those using the nearest ForCenS sample (Table S5). Thus, we only discuss the results based on the single nearest ForCenS sample.

### 2.7 Statistical analysis

For each species, the dependent response variable was the size distribution (natural logarithm of the 95th percentile of each population). The independent explanatory variables were the local mean annual SST, NPP, and relative abundance. Linear and quadratic relationships were considered between shell size and SST and NPP, as well as the interaction between SST and NPP. Model fit and selection was assessed using Akaike's information criterion corrected for small sample size (AICc); models within a difference of two AICc units (i.e., ∆AICc < 2) are equally plausible. Adjusted R squared (*R* ^2^_adj_) was calculated for each model using the R package *rsq* (version 1.0.1; Zhang, 2017). Visual inspection of the residual plots did not reveal any obvious deviations from homoscedasticity, except for *G. inflata* (Figure S1).

## 3 RESULTS

**FIGURE 4:**
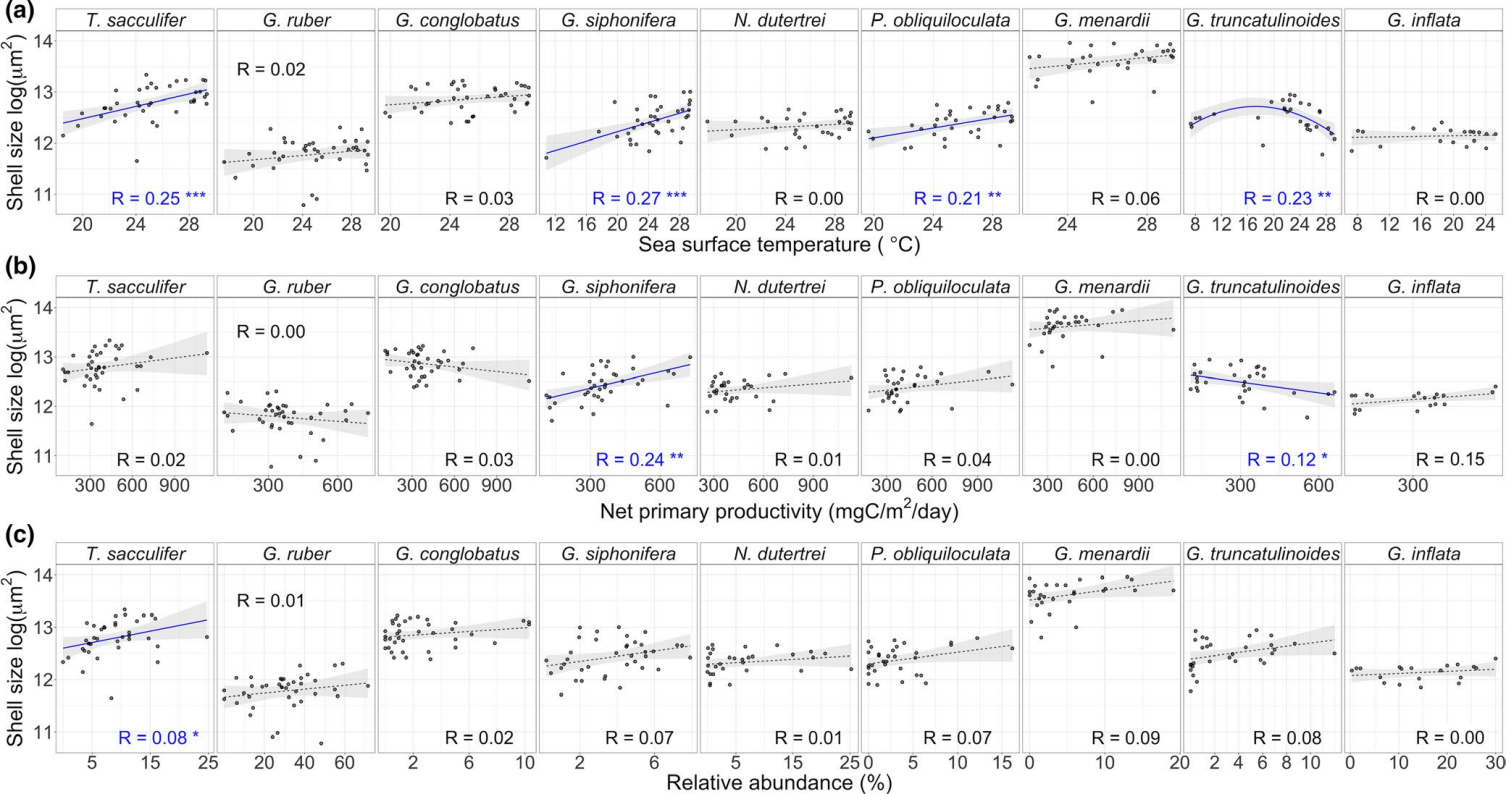
Size as a function of (a) mean annual sea‐surface temperature, (b) mean annual net primary productivity and (c) species' local relative abundance. Size was assessed as the cross‐sectional area of the shell, transformed by the natural logarithm, and represented by the 95th percentile of the population distribution. Solid blue lines show significant relationship whereas dashed gray lines nonsignificant; gray shades show standard error of the model. Most models are linear, except for the quadratic relationship between *Globorotalia* *truncatulinoides* size and temperature. The legend in each plot shows the adjusted R squared for each species. Significance codes: ***p < .001; **p < .01; *p < .05

Size variation within species of planktonic foraminifera is high (Figures 2 and 4) and can range over one order of magnitude among adults of the same species (e.g., from 150 to 1,500 μm in *G. menardii*, Figure 2). This high intraspecific variation relates differently to SST, NPP, and relative abundance for each species. When tested alone, SST explains most of the shell‐size variation within species, but only four of the nine studied species show a significant relationship between size and SST (Figure 4a). The tropical species *T. sacculifer*, *G. siphonifera,* and *P. obliquiloculata* show the expected positive linear relationship between SST and shell size, while the transitional *G. truncatulinoides* shows a quadratic relationship between shell size and SST. Results for these four species support previous observations that that planktonic foraminifera species are largest at their thermal optima (Schmidt, Renaud, et al., 2004). However, the remaining five species (namely *G. ruber*, *G. conglobatus*, *G. menardii*, *N. dutertrei,* and *G. inflata*) show no significant relationship between shell size and SST.

Six of the nine species studied are persistent (obligatory) photosymbiotic species (*G. siphonifera*, *T. sacculifer*, *G. ruber*, *G. conglobatus*, *N. dutertrei,* and *G. menardii*; Hemleben et al., 1989; Takagi et al., 2019). Of these, only *G. siphonifera* shows a significant, positive relationship between size and NPP (Figure 4b). The two facultative symbiotic species *P. obliquiloculata* and *G. inflata* (Takagi et al., 2019) show no detectable relationships between size and NPP. *G. truncatulinoides*, the only asymbiotic species in our study, shows a negative relationship between size and NPP (Figure 4b), contrary to the expected if species would grow more where more nutrients are available. Lastly, local relative abundance explains less size variation than SST and NPP for all species except *G. menardii* (Figure 4c). Only *T. sacculifer* shows a significant, positive relationship between shell size and relative abundance, but this relationship is weaker than with SST.

SST and NPP correlate (corr = 0.4, *p*‐value < .001), so their isolated effect on size might be confounding. For this reason, we considered all variables together in a model selection framework, including the interaction between SST and NPP. The explanatory power of SST, NPP, and relative abundance varies greatly among species (Table 2). SST explains 25% and 21% of the intraspecific size variation in *T. sacculifer* and *P. obliquiloculata*, respectively, and SST alone is the best explanatory model of *T. sacculifer*. SST and NPP together reach the highest predictability of size variation: 33% in *G. siphonifera* and 34% in *G. truncatulinoides*. SST and NPP are present in all the best explanatory models of *G. truncatulinoides* and *G. conglobatus*. The remaining four species, *G. ruber*, *N. dutertrei*, *G. menardii,* and *G. inflata*, include the null model (i.e., the sample mean) among the best explanatory models. Thus, size variation within these four species is poorly predicted by the three studied variables.

**TABLE 2:** Selection of the linear and quadratic models testing if planktonic foraminifera shell size can be predicted by mean annual sea‐surface temperature (sst linear effect; sst^2^ quadratic effect), mean annual net primary productivity (pp), their interaction (sst:pp), and/or species' relative abundance (abund)

Species	Exp. Var.	*df*	logLik	AICc	∆AICc	weight	*R* ^2^_adj_
*T. sacculifer*	sst	3	−7.04	20.79	0.00	0.40	**0.25**
*T. sacculifer*	sst2	4	−6.78	22.78	1.99	0.15	**0.24**
*G. ruber*	null	2	−12.45	29.22	0.00	0.18	0
*G. ruber*	sst + pp	4	−10.34	29.85	0.63	0.13	0.05
*G. ruber*	sst	3	−11.61	29.90	0.67	0.13	0.02
*G. ruber*	abund	3	−11.76	30.21	0.99	0.11	0.01
*G. ruber*	pp	3	−12.01	30.70	1.47	0.09	0
*G. ruber*	sst:pp	5	−9.45	30.71	1.49	0.09	0.07
*G. conglobatus*	sst:pp	5	5.29	1.30	0.00	0.28	0.19
*G. conglobatus*	sst + pp	4	3.40	2.40	1.10	0.16	0.13
*G. conglobatus*	sst2:pp	6	5.82	3.07	1.77	0.12	0.19
*G. siphonifera*	sst + pp	4	−2.22	13.69	0.00	0.28	**0.33**
*G. siphonifera*	sst	3	−4.24	15.20	1.51	0.13	**0.27**
*G. siphonifera*	sst:pp	5	−1.63	15.20	1.52	0.13	**0.33**
*N. dutertrei*	null	2	3.59	−2.73	0.00	0.22	0
*N. dutertrei*	pp	3	4.34	−1.76	0.98	0.13	0.01
*N. dutertrei*	abund	3	4.26	−1.60	1.13	0.12	0.01
*N. dutertrei*	sst	3	4.12	−1.32	1.41	0.11	0
*N. dutertrei*	sst2	4	5.25	−0.90	1.83	0.09	0.04
*P. obliquiloculata*	sst	3	1.89	3.08	0.00	0.31	**0.21**
*P. obliquiloculata*	sst + abund	4	2.50	4.47	1.39	0.15	**0.22**
*P. obliquiloculata*	sst + pp	4	2.26	4.97	1.89	0.12	**0.21**
*G. truncatulinoides*	sst2 + pp	5	2.99	6.51	0.00	0.39	**0.32**
*G. truncatulinoides*	sst2:pp	6	3.88	7.90	1.39	0.19	**0.34**
*G. menardii*	sst + abund	4	−0.43	10.53	0.00	0.23	0.15
*G. menardii*	abund	3	−2.08	11.11	0.58	0.17	0.09
*G. menardii*	sst	3	−2.53	12.02	1.50	0.11	0.06
*G. menardii*	null	2	−3.92	12.29	1.77	0.09	0
*G. inflata*	pp	3	11.11	−14.63	0.00	0.38	0.15
*G. inflata*	null	2	9.08	−13.41	1.22	0.21	0

Note

Size was assessed as the cross‐sectional area of the shell and represented by the 95th percentile of the population distribution. Columns: species, explanatory variables, degrees of freedom, log‐likelihood, Akaike's information criterion corrected for small sample size (AICc), AICc difference between models (∆AICc), model weight, and adjusted R squared (bold values: above 0.20). All models within two ∆AICc units are shown and considered equally plausible.

## 4 DISCUSSION

Our new morphometric dataset of species‐resolved planktonic foraminifera allowed us to explore the correlates of intraspecific size variation at a global scale. We tested the hypothesis that planktonic foraminifera species are largest under optimal environmental conditions (Hecht, 1976; Schmidt, Renaud, et al., 2004), identified using local SST, NPP, and relative abundance of species. We found a mixed picture: while *T. sacculifer*, *G. siphonifera*, *P. obliquiloculata,* and *G. truncatulinoides* reach larger sizes at specific environmental conditions, *G. ruber*, *G. conglobatus*, *N. dutertrei*, *G. menardii,* and *G. inflata* do not show statistically significant relationships between size and the studied variables (Figure 4, Table 2). Thus, our results provide weak support for the optimum‐size hypothesis.

Planktonic foraminifera shell size is the result of various processes such as respiration, calcification, symbiont photosynthesis, and feeding. Temperature influences the rates of these processes differently among species (Burke et al. 2018; Lombard, Erez, et al., 2009; Weinkauf, Kunze, Waniek, & Kucera, 2016). In addition, models show that planktonic foraminifera species have different temperature–growth relationships depending on their trophic strategy (Lombard, Labeyrie, Michel, Spero, & Lea, 2009). The species we studied vary along a trophic gradient from heterotrophy to persistent photosymbiosis (Takagi et al., 2019) and show variable temperature–size relationships (Figure 4a). However, species with the same trophic strategy did not show similar patterns (e.g., *T. sacculifer*, *G. conglobatus,* and *G. ruber*, Figure 4a). We also found weak evidence for links between shell size, open‐ocean NPP, and species' symbiotic strategy (Figure 4b). Other ecological interactions, such as predation, can also regulate the size distribution of zooplankton (top‐down control; Finlay, Beisner, Patoine, & Pinel‐Alloul, 2007) and might have different species‐specific effects in planktonic foraminifera (Burke & Hull, 2017). Given the various metabolic and ecological strategies of planktonic foraminifera, it is unlikely that temperature and NPP affect growth in similar ways across species.

The presence of cryptic genetic diversity can contribute to the high intraspecific variation found in our study. Some planktonic foraminifera species are complexes of lineages, which are genetically independent but morphologically similar (Darling & Wade, 2008). Cryptic species have been shown to occupy different niches and be endemic to particular ocean basins (Darling & Wade, 2008; De Vargas, Norris, Zaninetti, Gibb, & Pawlowski, 1999; Weiner et al., 2014). The global scale of our study likely includes cryptic diversity within our morphologically defined species. *T. sacculifer* and *G. conglobatus* are the only genetically homogeneous species in our study (Andre et al., 2013; Aurahs, Treis, Darling, & Kucera, 2011; Seears, Darling, & Wade, 2012). The other species comprise some level of cryptic diversity (Aurahs et al., 2011; Darling & Wade, 2008; De Vargas et al., 1999; Morard et al., 2011; Quillevere et al., 2011; Ujiie et al., 2012; Weiner, Weinkauf, Kurasawa, Darling, & Kucera, 2015; Weiner et al., 2014), except for *G. menardii*, whose genetic diversity has not yet been determined (Seears et al., 2012). The predictability of size variation in our study does not seem to relate directly to the cryptic diversity of species, as we find significant relationships between size and SST in genetically homogeneous and genetically diverse species (Figure 4a). Further, our results suggest that the distinct genetic types within *G. siphonifera*, *P. obliquiloculat*a, and *G. truncatulinoides* might have similar growth responses to SST variation.

The Buckley Collection (Rillo et al., 2016) contains samples from historical expeditions that collected marine sediments using devices such as a dredge. These devices potentially disturb the ocean floor surface and can recover a mix of Holocene (surface) and deeper, older sediments (Rillo, Kucera, Ezard, & Miller, 2019). This source of bias is inherent to this museum collection, as it includes samples from pioneering marine expeditions such as HMS *Challenger*. Nevertheless, it can potentially increase the size variation observed because of the temporal mixing of samples. We assessed this bias by rerunning the analysis without the samples recovered using dredges or grabbers (six in total, see Table S1). Most model results were unchanged except for two species: The best models of *G. conglobatus* include the null model and *G. inflata* exclude the null model (Table S6). Overall, the general pattern remains: Intraspecific size variation in planktonic foraminifera cannot be explained consistently for all species.

The local relative abundance of a species was in general a poor predictor of its size variation (Figure 4c, Table 2), contrary to the idea that planktonic foraminifera species are largest where they are most common (Hecht, 1976). The reason we find a weak relationship between size and local relative abundance could be related to using abundance data from a different source (the ForCenS database, see Section 2.6). We assessed the robustness of our results by testing the optimum‐size hypothesis on a more uniform, but smaller, dataset: the ten resampled bulk samples used for the bias analysis (Figures 1a and 3, Table S3). Following the same methodology as before, we measured shell area and calculated species' relative abundances for each of the ten assemblages, so that the same individuals were used to extract abundance and size data. 65 populations of 20 species were then used to test if population shell size could be predicted by relative abundance using a linear‐mixed effect model with species as random effects. The results showed no significant relationship between size variation and relative abundance (chi‐square test, *χ* ^2^ = 2.18, *p*‐value = .14, Table S4), supporting our previous findings using the larger Buckley Collection data (Table 2, Figure 4c).

The use of relative, instead of absolute, abundance as an indicator of planktonic foraminifera environmental optima (e.g., Hecht, 1976; Kucera, 2007; Schmidt, Renaud, et al., 2004; this study) can be misleading. A species can reach high relative abundance because it is better able to tolerate stress than other species. As a result of stress, the total, absolute abundances of species will be low, but the most tolerant species will reach high *relative* abundances even under suboptimal conditions (Hecht, 1976). Moreover, when a species is less abundant (or longer‐lived) than other species in the local plankton community, its relative abundance in the sediment will be strongly influenced by the more abundant (or shorter‐lived) co‐occurring species. In fact, studies using absolute abundance data from plankton nets (Aldridge, Beer, & Purdie, 2012; Beer, Schiebel, & Wilson, 2010) and sediment traps (Weinkauf et al., 2016) failed to find support for a positive relationship between shell size and absolute abundance in *G. ruber* and *Globigerina bulloides*. Ideally, the environmental optimum of a planktonic foraminifera species would be determined based on its absolute abundances across its geographical range, independently of the abundances of other species.

5 | CONCLUSION

The idea that planktonic foraminifera species are largest at their environmental optima is based on basin‐ or species‐specific studies (Hecht, 1976; Kahn, 1981; Kennett, 1976; Malmgren & Kennett, 1976, 1977; Moller et al., 2013) and the analysis of a subset of samples from the assemblage‐level study of Schmidt, Renaud, et al. (2004). By sampling broader geographical ranges with larger sample sizes compared to previous studies, we found weak support for the optimum‐size hypothesis among the nine studied species. Most of the within‐species size variation was not explained by the tested environmental parameters (Figure 4). As we move toward higher qualitative and quantitative resolution of measurements (e.g., Hsiang et al., 2019), we expect to increasingly uncover the ecological and morphological diversity within and among planktonic foraminifera species.

## CONFLICT OF INTEREST

None declared.

## AUTHOR CONTRIBUTION

**Marina C. Rillo:** Conceptualization (lead); Data curation (lead); Formal analysis (lead); Funding acquisition (supporting); Investigation (lead); Methodology (lead); Project administration (lead); Validation (lead); Visualization (lead); Writing‐original draft (lead); Writing‐review & editing (lead). **C. Giles Miller:** Conceptualization (equal); Data curation (lead); Methodology (equal); Project administration (equal); Resources (lead); Supervision (equal); Writing‐review & editing (equal). **Michal Kucera:** Conceptualization (equal); Funding acquisition (supporting); Investigation (equal); Methodology (equal); Project administration (equal); Supervision (equal); Writing‐review & editing (equal). **Thomas H. G. Ezard:** Conceptualization (equal); Formal analysis (equal); Funding acquisition (lead); Investigation (equal); Methodology (equal); Resources (lead); Supervision (equal); Writing‐review & editing (lead).

## Supporting information

Appendix S1

## Data Availability

The data and the R code used to produce the analyses are available from the NHM Data Portal: https://doi.org/10.5519/0056541. Specimens' images can be found at https://doi.org/10.5519/0035055 (via the ZF number) and as part of the Endless Forams project (http://endlessforams.org).
