# Supplementary material for: Intraspecific size variation in planktonic foraminifera cannot be consistently predicted by the environment
Source: Ecol Evol. 2020 Sep 28;10(20):11579–90. doi: 10.1002/ece3.6792 (PMC7593196; doi:10.1002/ece3.6792)

Supplementary Material

Intraspecific size variation in planktonic foraminifera cannot be consistently predicted by the environment

Rillo, M.C.; Miller, C.G.; Kucera, M. & Ezard, T.H.G.

Ecology and Evolution (in press)

https://doi.org/10.1002/ece3.6792

The data and the R code used to produce the analyses are available from the NHM Data Portal: https://doi.org/10.5519/0056541, including Table S1 and Table S3 as spreadsheets. Specimens' images can be found at https://doi.org/10.5519/0035055 (via the ZF number) and as part of the Endless Forams project (http://endlessforams.org).

Table S1: Samples from the Henry Buckley Collection of Planktonic Foraminifera at The Natural History Museum, London (NHMUK) used in the morphometric analysis. Columns: NHMUK Internal Record Number of the sediment in the Ocean-Bottom Deposits Collection (OBD IRN); name of the vessel that collected the sample; year the sample was collected; latitude (Lat) and longitude (Long) given in decimal degrees; sea surface temperature (SST) in Celsius degrees; water depth in meters; sampling method used in the historical expedition; depth below the seafloor (Dbsf) sampled in centimetres; number of planktonic foraminifera specimens N(ind) and species N(ssp) measured at each site.

OBD IRN	Vessel	Year	Lat	Long	SST	Depth(m)	Sampling	Dbsf(cm)	N(ind)	N(ssp)
31945	CS Britannia	1899	39.21	-70.24	19.25	-2731	Sounding	surface	65	1
34297	CS Buccaneer	1886	-0.03	-15.94	26.3	-3226	Sounding	surface	57	1
30724	HEMS Mabahiss	1934	7.19	63.04	28.38	-4346	Core	4-5.5	53	7
35818	HMIMS Investigator	1906	20.44	68.84	27.24	-2680	Sounding	surface	56	5
17229	HMNZS Lachlan	1955	-33.88	173.83	17.8	-2464	Worsel sampler	surface	106	8
17262	HMNZS Pukaki	1957	-53.63	169.87	7.95	-746	Dietz grab	surface	52	2
33286	HMS Challenger	1873	24.33	-24.47	23.16	-5153	Sounding	surface	46	1
32657	HMS Challenger	1874	-50.02	123.07	9.32	-3976	Sounding	surface	11	1
33668	HMS Challenger	1875	-0.7	147	29.4	-2213	Sounding	surface	95	7
34607	HMS Challenger	1875	-33.7	-78.3	16.79	-3798	Sounding	surface	8	1
34991	HMS Challenger	1876	-21.25	-14.03	23.61	-3740	Dredge	surface	181	8
34671	HMS Egeria	1887	-19.57	64.63	24.83	-2708	Sounding	surface	66	8
34676	HMS Egeria	1887	-23.23	56.3	25.14	-4646	Sounding	surface	73	9
34678	HMS Egeria	1887	-29.93	54.1	21.13	-4211	Sounding	surface	63	8
34993	HMS Egeria	1889	-15.65	-179.06	28.05	-2519	Sounding	surface	124	8
35238	HMS Egeria	1894	7.08	73.8	28.57	-2658	Sounding	surface	66	7
16621	HMS Enterprise	1962	30.9	-78.68	26.63	-821	Dredge	surface	35	1
36043	HMS Penguin	1891	-28.01	112.46	21.94	-1206	Sounding	surface	189	9
36053	HMS Penguin	1891	-26.94	111.18	22.66	-3350	Sounding	surface	203	9
36057	HMS Penguin	1891	-24.89	110.39	22.83	-3829	Sounding	surface	193	9
36361	HMS Penguin	1896	-10.21	178.01	28.91	-4844	Sounding	surface	123	8
36515	HMS Penguin	1897	-9.68	-174.62	28.05	-4057	Sounding	surface	71	6
36683	HMS Penguin	1897	1.21	-161.84	27.32	-4634	Sounding	surface	47	8
36704	HMS Penguin	1897	-13.17	-175.69	28.05	-3952	Sounding	surface	111	7
37130	HMS Sealark	1905	-8.42	65.63	28.35	-3694	Sounding	surface	78	7
37148	HMS Sealark	1905	-7.59	61.48	28.06	-3507	Sounding	surface	87	8
37149	HMS Sealark	1905	-2.7	67.38	28.95	-3594	Sounding	surface	119	7
37190	HMS Sealark	1905	-12.12	64.12	27.16	-3322	Sounding	surface	72	7
37299	HMS Serpent	1868	18.63	69.17	27.85	-3261	Sounding	surface	9	1
38482	HMS Waterwitch	1895	-40.45	49.82	7.78	-3780	Sounding	surface	39	2
17031	RNZFA Tui	1956	-39.77	167.75	16.04	-1137	Dietz grab	surface	71	4

17240	RNZFA Tui	1956	-28.88	170	22.18	-3021	Dietz grab	surface	165	8
17273	RNZFA Tui	1958	-20.95	-175.23	25.66	-869	Cone dredge	surface	40	6
16657	RV Argo	1960	-16.42	66.03	26.2	-2810	Core	4-9	60	8
16365	RV Horizon	1953	-19.48	-173.73	25.66	-4347	Gravity core	5-10	43	4
16640	RV Horizon	1953	-13.09	-124.28	26.67	-3456	Gravity core	4-8	58	7
16641	RV Horizon	1953	-14.27	-120.68	25.97	-3617	Gravity core	4-8	41	6
16642	RV Horizon	1953	-15.22	-117.51	25.97	-3641	Gravity core	1-4	32	5
16656	RV Horizon	1958	-23.61	-118.22	23.98	-3362	Gravity core	7-11	48	5
8754166	RV Maria Paolina G	1970	35.68	-4.08	18.17	-1500	Sphincter core	0-5	40	3
16645	RV Spencer F. Baird	1957	5.43	-131.32	27.65	-3415	Gravity core	7-10	51	6
16646	RV Spencer F. Baird	1957	-26.32	-147.12	21.85	-2312	Gravity core	3-7	54	6
16647	RV Spencer F. Baird	1957	-46.75	-123	9.91	-4030	Gravity core	6-11	53	2
16648	RV Spencer F. Baird	1957	-48.48	-113.28	8.06	-2677	Gravity core	0-8	41	2
16649	RV Spencer F. Baird	1957	-43.72	-107.6	10.99	-3141	Gravity core	3-7	17	3
16370	RV Spencer F. Baird	1958	-11.7	-109.72	25.27	-3296	Gravity core	0-3	1	1
16650	RV Spencer F. Baird	1958	-18.33	-79.34	20.07	-3157	Gravity core	8-12	49	7
16651	RV Spencer F. Baird	1958	-27.93	-106.92	21.45	-3039	Gravity core	8-15	67	6
16652	RV Spencer F. Baird	1958	-27.15	-109.83	21.45	-2819	Gravity core	10-14	51	5
16653	RV Spencer F. Baird	1958	-14.73	-112.1	24.9	-3034	Gravity core	12-15	69	7
16654	RV Spencer F. Baird	1958	-9.88	-110.68	25.26	-2712	Gravity core	5-9	46	7
17162	RV Vema	1959	28.4	-77.93	24.89	-1004	Piston core	0-2	136	8
17359	RV Vema	1959	-9.75	-34.4	27.14	-4123	Piston core	5-6	86	6

Table S2: Dissolution analysis: linear mixed-effects model (LMER, ANOVA), using size variation (95th percentile of the population) as the response variable, species as the random effects and either a null model (H0) or water depth (H1) as the explanatory variable. Columns: model explanatory variables (fixed effects), degrees of freedom, Akaike Information Criterion, log-likelihood, model deviance, chi-squared, p-value, marginal R squared.

Exp. Var.	Df	AIC	logLik	Dev.	Chi-sq	p-value	R_m^2
null	5	151.67	-70.84	141.67	NA	NA	0
water depth	6	151.75	-69.87	139.74	1.93	0.16	0.00

Museum bias analysis methods: to assess the size bias in the Buckley Collection, we re-sampled ten bulk sediments of the NHMUK Ocean-Bottom Deposits Collection (OBD) Collection, from which the Buckley Collection was created (Fig. 1a, Table S3). Samples were chosen to encompass different oceans, latitudes and marine expeditions; however, the final choice also depended on the availability of bulk sediment samples in the OBD Collection. Half of the amount available in the OBD containers was further split into two equal parts, leaving an archive sample and a sample to be processed. The sample processing consisted of weighing, wet washing over a 63µm sieve and drying in a 60°C oven. The residues were further dry sieved over a 150µm sieve and the coarser fraction was split with a microsplitter as many times as needed to produce a representative aliquot containing around 300 planktonic foraminifera specimens (see Al-Sabouni et al. 2007). All specimens in each of the nine final splits were identified by MCR and MK under a stereomicroscope to species level, resulting in a total of 2,611 individuals belonging to 31 species (see also Rillo et al. 2019). We mounted species-specific slides from the re-sampled samples, calculated the relative abundance of each species in each sample, and then extracted shell size data in the same way as for the slides of the Buckley Collection. Only the sizes of species also present in the Buckley Collection samples were measured, resulting in 1824 specimens' shell sizes from 20 species (Table S3). See Methods section in the main text for further information.

Table S3: Bias analysis: samples re-sampled from the Ocean-Bottom Deposits (OBD) Collection at the Natural History Museum, London (NHMUK) used in the museum collection size bias analysis. Columns: NHMUK Internal Record Number of the sediment in the OBD Collection; name of the Vessel that collected the sample; latitude (Lat) and longitude (Long) given in decimal degrees; water depth in meters; sampled mass in grams; number of times sediment was split N(splits) to achieve around 300 specimens; number of planktonic foraminifera specimens N(ind) and species N(ssp) measured in each re-sampled sample.

OBD_IRN	Vessel	Lat	Long	Depth	Mass (g)	N(splits)	N(ind)	N(ssp)
32657	HMS *Challenger*	-50.01667	123.06667	-3976	0.187	5	318	7
34991	HMS *Challenger*	-21.25	-14.03333	-3740	9.35	7	265	21
33668	HMS *Challenger*	-0.7	147	-2213	1.98	7	331	16
33286	HMS *Challenger*	24.33333	-24.46667	-5153	2.73	5	260	18
34671	HMS *Egeria*	-19.56667	64.63333	-2708	1.23	5	376	24
34993	HMS *Egeria*	-15.65	-179.0625	-2519	2.42	8	300	13
36053	HMS *Penguin*	-26.93667	111.18167	-3350	1.49	5	279	18
37148	HMS *Sealark*	-7.59167	61.48333	-3507	2.86	8	305	18
38482	HMS *Waterwitch*	-40.45	49.81667	-3780	1.51	6	177	11
14609	Alpha 6	85.25	-167.9	-1774	0.57	4	226	1
						**TOTAL**	**2837**	**31**

Linear mixed-effects regression using the bias analysis data: using the re-sampled populations described above, we tested whether relative abundance variation significantly explains population shell-size variation. Since the re-sampled data includes only ten samples (Fig. 1a), there is not enough data to run species-specific generalised linear models (GLM). Instead, we used linear mixed-effect models (LMER). The log-transformed 95th percentiles of the population shell-size distributions were modelled as the response variable, and the independent fixed effect was the species' relative abundance in the re-sampled sample. Species were modelled as random effects, allowing for random intercepts and slopes (i.e., the intercept and slope of the relationship between shell size and the relative abundance may vary among species). We used the Likelihood Ratio Test (LRT) to compare the likelihood of the fixed effect. We calculated the LRT between the models with and without the effect. Significance of each fixed effect was given through the LRT. Marginal R^2 (R_m^2), which is associated with the fixed effects, was calculated for each LMER model.

Table S4: Bias analysis: linear mixed-effects model (LMER, ANOVA) using the re-sampled data, and size variation (log-transformed 95th percentile of the population) as the response variable, species as the random effects (r.e.) and either a null model (H0) or relative abundance (H1) as the explanatory variable. Columns: model explanatory variables (fixed effects), degrees of freedom, Akaike Information Criterion, log-likelihood, model deviance, chi-squared, p-value, marginal R squared.

Exp. Var.	Df	AIC	logLik	Dev.	Chi-sq	p-value	R_m^2
null	5	130.20	-60.01	120.20	NA	NA	0
relative abundance	6	130.02	-59.01	118.02	2.18	0.14	0.07

Table S5: Analysis considering relative abundance samples within a 300 km distance: model selection of the linear and quadratic models testing if planktonic foraminifera shell size (represented by the 95th percentile of each population size distribution) can be predicted by mean annual sea-surface temperature (sst linear effect, sst^2 quadratic effect), mean annual net-primary productivity (npp) and/or species' relative abundance (abund, calculated as the median relative abundance of all samples within a 300 km distance of each morphometric sample). A model including the interaction between sst and npp (sst : npp) was also considered. Columns: species, explanatory variables, degrees of freedom, log-likelihood, Akaike Information Criterion corrected for small sample size (AICc), AICc difference between models (ΔAICc), model weight, adjusted R squared (values above 0.15 are in bold). All models within two ΔAICc units are shown and considered equally plausible.

Species	Exp. Var.	df	logLik	AICc	ΔAICc	weight	R_{adj}^2
T. sacculifer	sst	3	-7.04	20.79	0	0.38	0.25
T. sacculifer	sst2	4	-6.78	22.78	1.99	0.14	0.24
G. ruber	null	2	-12.45	29.22	0	0.18	0
G. ruber	sst + pp	4	-10.34	29.85	0.63	0.13	0.05
G. ruber	sst	3	-11.61	29.9	0.67	0.13	0.02
G. ruber	abund	3	-11.69	30.06	0.84	0.12	0.01
G. ruber	pp	3	-12.01	30.7	1.47	0.09	0
G. ruber	sst : pp	5	-9.45	30.71	1.49	0.08	0.07
G. conglobatus	sst : pp	5	5.29	1.3	0	0.27	0.19
G. conglobatus	sst + pp	4	3.4	2.4	1.1	0.16	0.13
G. conglobatus	sst2 : pp	6	5.82	3.07	1.77	0.11	0.19
G. siphonifera	sst + pp	4	-2.22	13.69	0	0.29	0.33
G. siphonifera	sst	3	-4.24	15.2	1.51	0.14	0.27
G. siphonifera	sst : pp	5	-1.63	15.2	1.52	0.14	0.33
N. dutertrei	null	2	3.59	-2.73	0	0.22	0
N. dutertrei	pp	3	4.34	-1.76	0.98	0.13	0.01
N. dutertrei	abund	3	4.17	-1.42	1.31	0.11	0
N. dutertrei	sst	3	4.12	-1.32	1.41	0.11	0
N. dutertrei	sst2	4	5.25	-0.9	1.83	0.09	0.04
P. obliquiloculata	sst	3	1.89	3.08	0	0.35	0.21
P. obliquiloculata	sst + pp	4	2.26	4.97	1.89	0.13	0.21
G. menardii	sst	3	-2.53	12.02	0	0.18	0.06
G. menardii	sst + abund	4	-1.29	12.26	0.23	0.16	0.1
G. menardii	null	2	-3.92	12.29	0.27	0.16	0
G. menardii	abund	3	-3.02	13.01	0.99	0.11	0.02
G. menardii	pp	3	-3.48	13.92	1.9	0.07	-0.01
G. truncatulinoides	sst2 + pp	5	2.99	6.51	0	0.36	0.32
G. truncatulinoides	sst2 : pp	6	3.88	7.9	1.39	0.18	0.34
G. inflata	pp	3	11.11	-14.63	0	0.38	0.15
G. inflata	null	2	9.08	-13.41	1.22	0.21	0

Table S6: Analysis excluding historical samples collected by dredging the ocean floor, namely samples collected by HMNZS *Pukaki*, HMS *Challenger* (dredge only), HMS *Enterprise* and RNZFA *Tui* (see Table S1). Columns and variables: see description of Table S5.

Species	Exp. Var.	df	logLik	AICc	ΔAICc	weight	R_{adj}^2
T. sacculifer	sst	3	-5.29	17.34	0	0.32	0.3
T. sacculifer	sst + pp	4	-4.76	18.81	1.47	0.15	0.3
T. sacculifer	sst2	4	-4.88	19.05	1.71	0.14	0.3
G. ruber	null	2	-12.4	29.16	0	0.21	0
G. ruber	sst	3	-11.5	29.75	0.6	0.16	0.02
G. ruber	sst + pp	4	-10.53	30.35	1.19	0.12	0.04
G. ruber	abund	3	-11.91	30.57	1.41	0.11	0
G. ruber	pp	3	-12.12	30.98	1.82	0.09	-0.01
G. conglobatus	sst + pp	4	3.71	1.87	0	0.15	0.11
G. conglobatus	sst : pp	5	5.06	1.88	0.02	0.15	0.15
G. conglobatus	sst + abund	4	3.48	2.33	0.46	0.12	0.1
G. conglobatus	sst	3	1.93	2.89	1.02	0.09	0.05
G. conglobatus	sst + abund + pp	5	4.36	3.28	1.41	0.07	0.12
G. conglobatus	null	2	0.45	3.45	1.59	0.07	0
G. conglobatus	abund	3	1.61	3.54	1.67	0.06	0.03
G. conglobatus	sst2 : pp	6	5.63	3.65	1.78	0.06	0.15
G. siphonifera	sst + pp	4	-2.06	13.51	0	0.28	0.35
G. siphonifera	sst	3	-4.09	14.98	1.47	0.13	0.29
G. siphonifera	sst : pp	5	-1.42	14.98	1.47	0.13	0.35
N. dutertrei	pp	3	6.51	-6.06	0	0.24	0.07
N. dutertrei	null	2	4.93	-5.4	0.66	0.17	0
N. dutertrei	sst	3	5.52	-4.07	1.99	0.09	0
P. obliquiloculata	sst	3	2.31	2.28	0	0.35	0.18
P. obliquiloculata	sst + abund	4	2.79	3.96	1.68	0.15	0.18
G. menardii	abund	3	-1.68	10.36	0	0.23	0.08
G. menardii	sst + abund	4	-0.59	10.92	0.56	0.17	0.11
G. menardii	null	2	-3.34	11.16	0.81	0.15	0
G. menardii	sst	3	-2.5	12	1.64	0.1	0.02
G. truncatulinoides	sst2 + pp	5	2.16	8.84	0	0.24	0.31
G. truncatulinoides	sst2	4	0.47	9.06	0.23	0.21	0.24
G. truncatulinoides	sst2 + abund	5	1.62	9.92	1.08	0.14	0.28
G. inflata	pp	3	10.03	-12.07	0	0.53	0.26

Fig. S1: Residual plots of linear models per species. Models: null, abund (relative abundances), sst (mean annual sea surface temperature), and pp (mean annual net primary productivity).

Trilobatus sacculifer

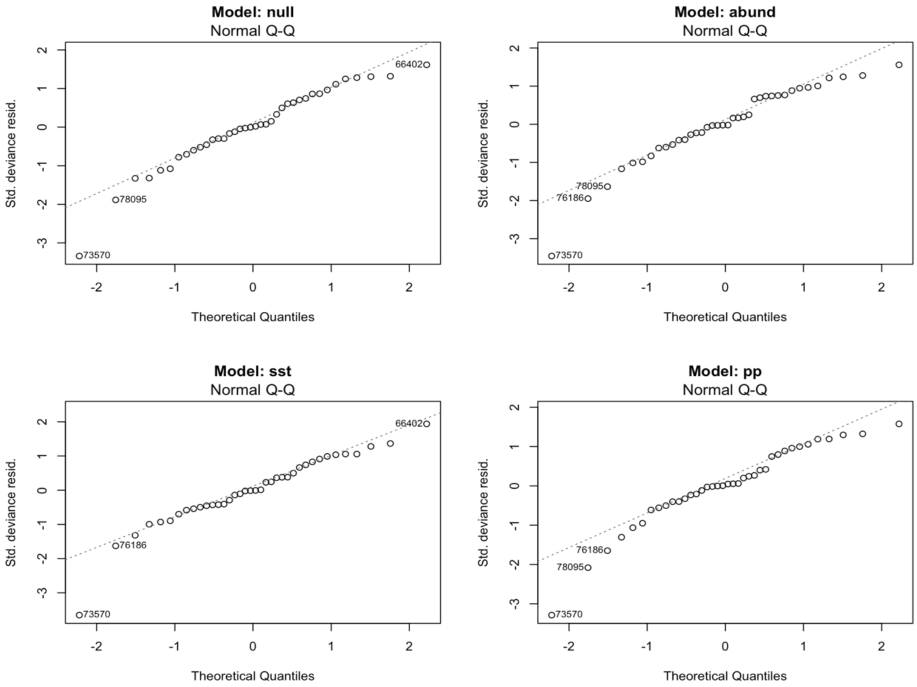

Globigerinoides ruber

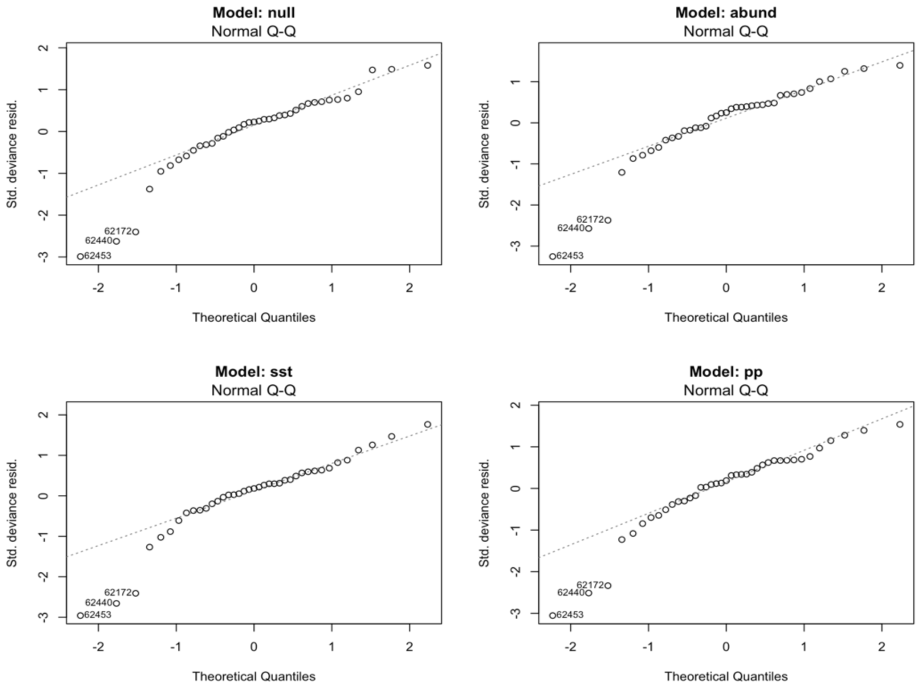

Globigerinoides conglobatus

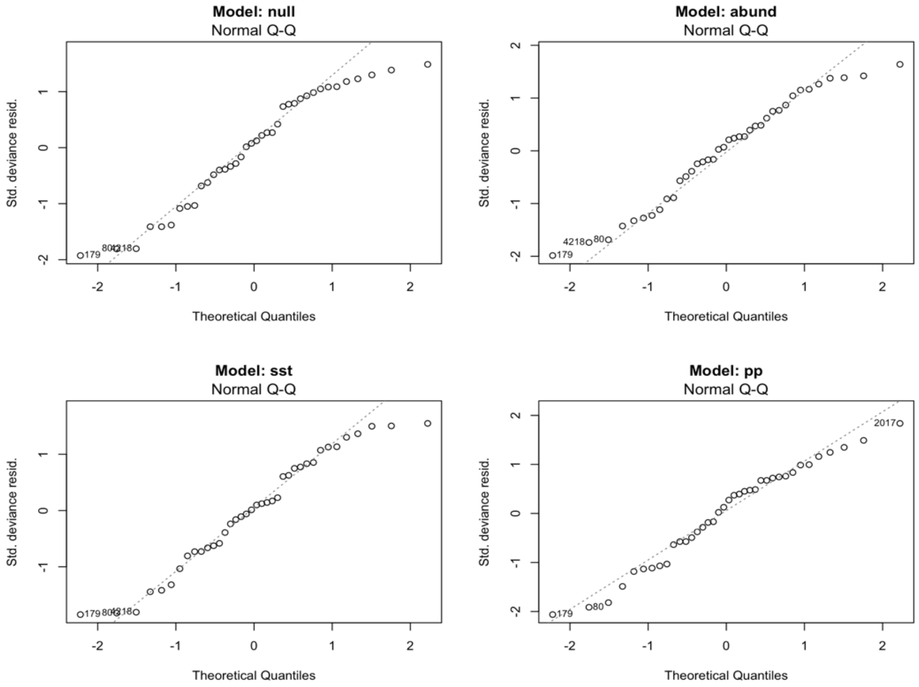

Globigerinella siphonifera

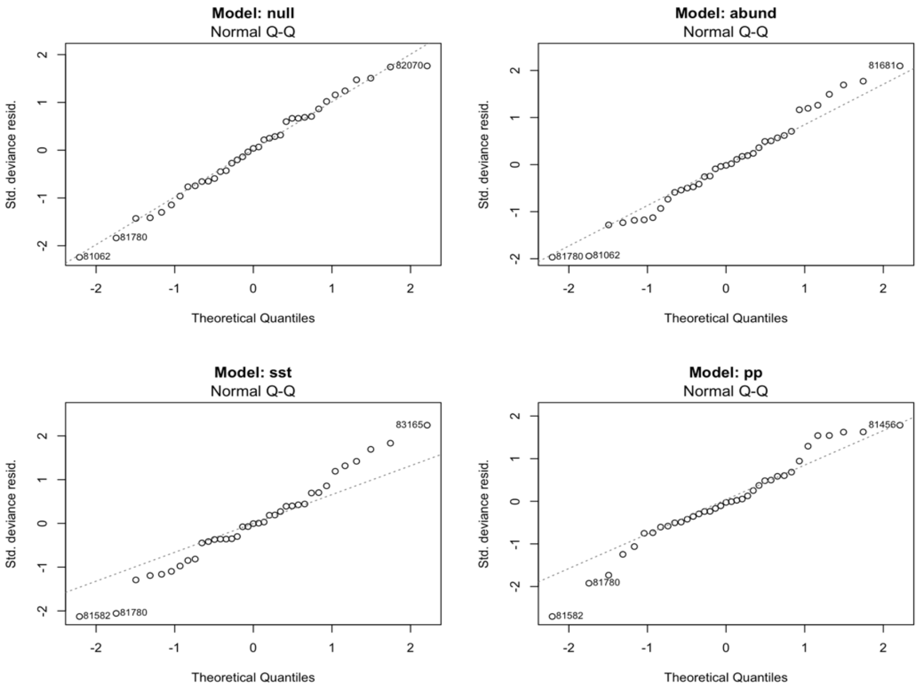

Neogloboquadrina dutertrei

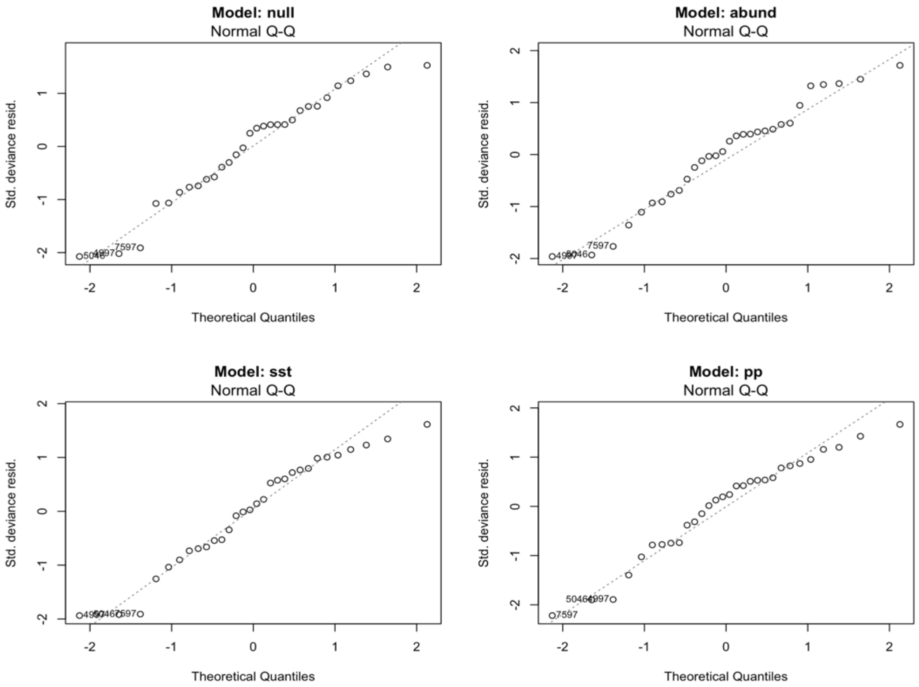

Pulleniatina obliquiloculata

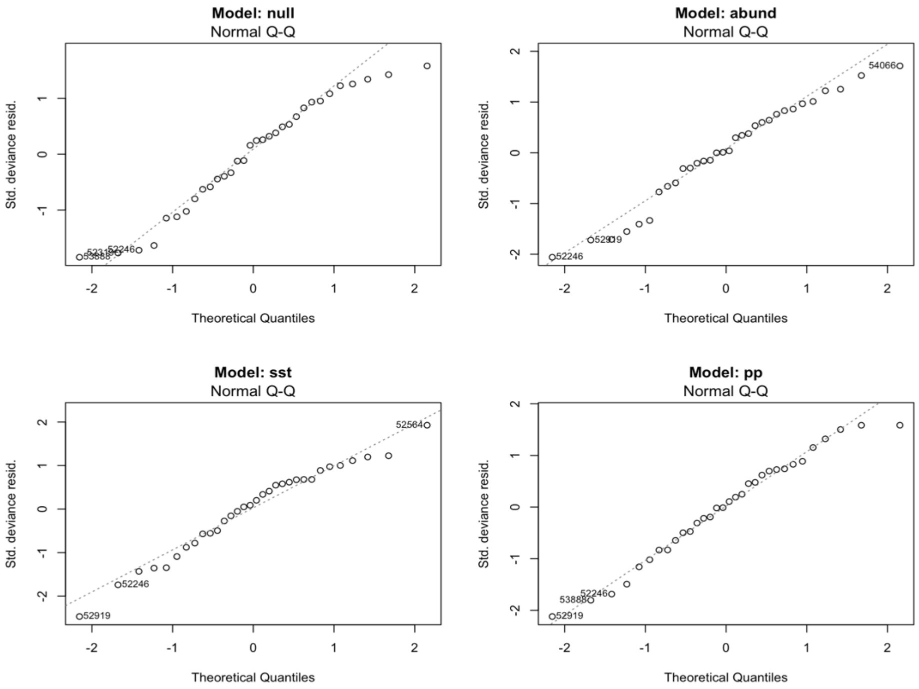

Globorotalia menardii

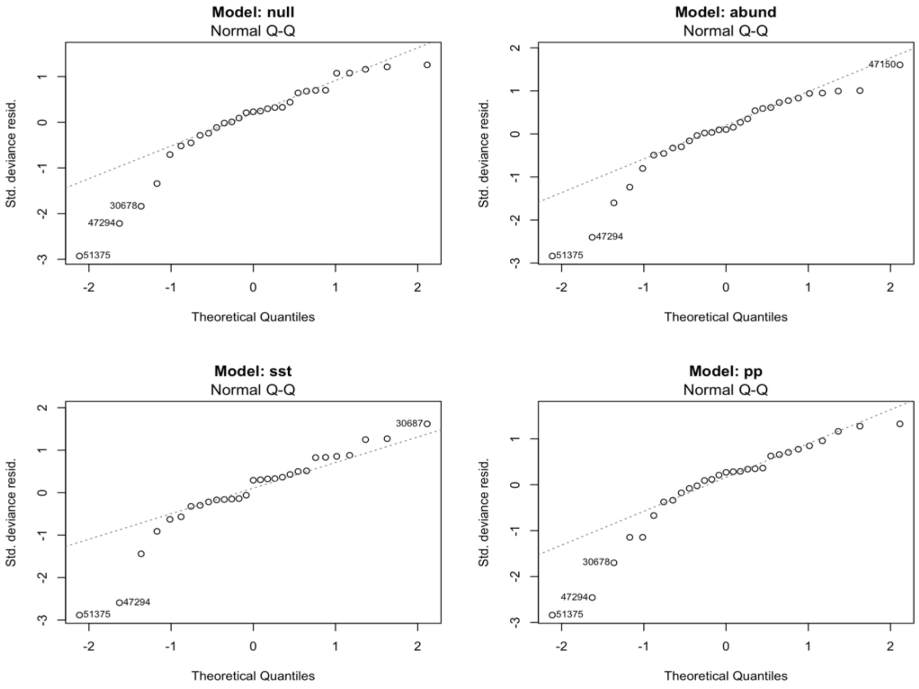

Globorotalia truncatulinoides

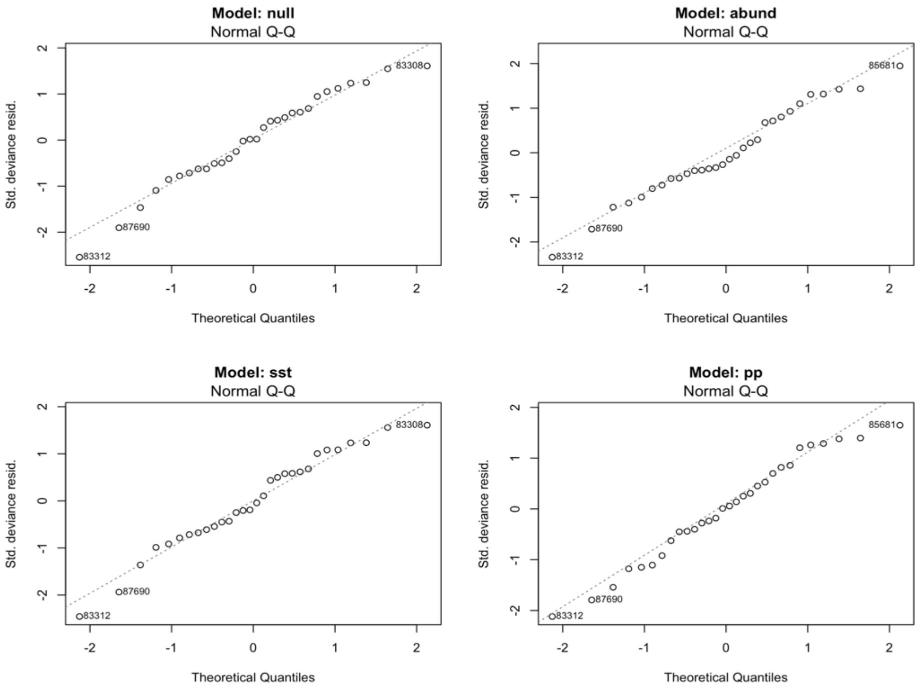

Globoconella inflata

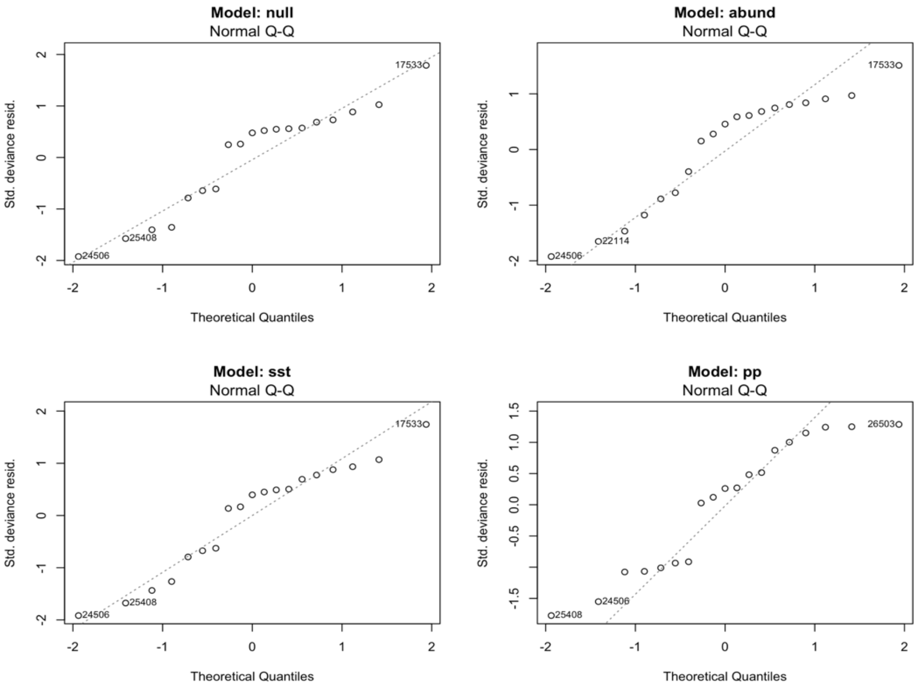

Supplement: Supplementary file 1 — Appendix S1 [file ECE3-10-11579-s001.pdf]
